# Sodium Levels of Processed Meat in Australia: Supermarket Survey Data from 2010 to 2017

**DOI:** 10.3390/nu10111686

**Published:** 2018-11-05

**Authors:** Emalie Sparks, Clare Farrand, Joseph Alvin Santos, Briar McKenzie, Kathy Trieu, Jenny Reimers, Chelsea Davidson, Claire Johnson, Jacqui Webster

**Affiliations:** 1 The George Institute for Global Health, The University of New South Wales, Sydney, NSW 2006, Australia; cfarrand@georgeinstitute.org.au (C.F.); jsantos@georgeinstitute.org.au (J.A.S.); bmckenzie@georgeinstitute.org.au (B.M.); ktrieu@georgeinstitute.org.au (K.T.); cjohnson@georgeinstitute.org.au (C.J.); jwebster@georgeinstitute.org.au (J.W.); 2 Victorian Health Promotion Foundation (VicHealth), 15-31 Pelham Street, Carlton, VIC 3053, Australia; jreimers@vichealth.vic.gov.au; 3 Heart Foundation, Level 2, 850 Collins Street, Docklands, VIC 3008, Australia; chelsea.davidson@heartfoundation.org.au

**Keywords:** sodium levels, processed meat, food reformulation, Australia

## Abstract

High sodium intake increases blood pressure and consequently increases the risk of cardiovascular diseases. In Australia, the best estimate of sodium intake is 3840 mg sodium/day, almost double the World Health Organization (WHO) guideline (2000 mg/day), and processed meats contribute approximately 10% of daily sodium intake to the diet. This study assessed the median sodium levels of 2510 processed meat products, including bacon and sausages, available in major Australian supermarkets in 2010, 2013, 2015 and 2017, and assessed changes over time. The median sodium content of processed meats in 2017 was 775 mg/100 g (interquartile range (IQR) 483–1080). There was an 11% reduction in the median sodium level of processed meats for which targets were set under the government’s Food and Health Dialogue (*p* < 0.001). This includes bacon, ham/cured meat products, sliced luncheon meat and meat with pastry categories. There was no change in processed meats without a target (median difference 6%, *p* = 0.450). The new targets proposed by the current government’s Healthy Food Partnership capture a larger proportion of products than the Food and Health Dialogue (66% compared to 35%) and a lower proportion of products are at or below the target (35% compared to 54%). These results demonstrate that voluntary government targets can drive nutrient reformulation. Future efforts will require strong government leadership and robust monitoring and evaluation systems.

## 1. Introduction

Sodium is a chemical element required by the body for many physiological functions including maintenance of blood volume and pressure. However, excess dietary sodium intake, increases blood pressure and consequently increases the risk of cardiovascular diseases (CVDs) [1]. CVDs are the leading cause of death in Australia, accounting for 32% of all deaths [2]. Sodium is found naturally in some foods, however most sodium consumed is added to foods either by a manufacturer or consumer in the form of sodium chloride, more commonly known as salt [3]. Correspondingly, reducing salt intake to less than 5 g/day (2000 mg sodium), as recommended by the World Health Organization (WHO) [4], is an effective way to reduce the risk of CVDs through lowering blood pressure, as well as other adverse health implications associated with high salt intakes including chronic kidney disease, obesity, gastric cancer and liver diseases [5,6,7,8]. As such, Australia has committed to reducing population salt intake by 30% by 2025, alongside all WHO member states, to reduce premature death from non-communicable diseases (NCDs) [9].

Worldwide, populations are consuming in excess of 5 g of salt (2000 mg sodium) per day [10], and in Australia the best estimate is 9.6 grams salt per day [11]. While there is no national salt reduction strategy in Australia, the federal government, many state governments and numerous non-government organizations are currently involved in salt-reduction initiatives. In 2009, the Australian government established the Food and Health Dialogue (FHD), with a plan for sodium reduction in nine priority food categories [12]. The FHD has since been succeeded by the Healthy Food Partnership (HFP) [13], which in 2018 published draft sodium targets for 30 food categories, as well as proposing sugar and saturated fat targets for selected food categories. It has been suggested that inadequacies in governance and implementation may have hindered the FHD’s impact, and that the key to successful implementation of the HFP will be strong leadership by the government, along with robust monitoring and evaluation systems [14].

Processed meats were identified as a priority food category for product reformulation by both the FHD and HFP [12,13]. The most recent Australian Health Survey estimated processed meats contribute approximately 10% of daily sodium intake to the Australian diet, and make up an estimated 20% of the population’s meat intake [15]. In addition, almost 30% of the population consumed processed meats on the day of the survey; with 12% consuming ham, 6% consuming sausages and 5% consuming bacon [15]. Overall, the most commonly consumed processed meats were sausages (38%), ham and bacon (36%), luncheon meats (11%), salami (7%), frankfurts (4%) and nuggets (<1%) [15].

The Victorian Health Promotion Foundation (VicHealth) Salt Reduction Partnership Group was established to reduce salt intake in Victoria, with the aim of achieving a 1 g reduction by June 2020 [16,17]. A main component of the strategy is to monitor sodium levels in the food supply and use the results to engage and support the food industry in salt reduction reformulation [17]. As part of the Partnership strategy, this study aimed to determine the changes in sodium levels of processed meats sold between 2010 and 2017, and determine whether target setting drove processed meat product reformulation.

## 2. Materials and Methods

A systematic survey of the sodium content of products in four major supermarkets in Australia (Coles, Woolworths, IGA and Aldi) was conducted each year as part of The George Institute for Global Health’s FoodSwitch database protocol, which includes a quality-assurance procedure [18]. The data were obtained from the nutrition information panel, and included manufacturer name, brand name, product name, package size, serving size, and sodium (mg/100 g food, mg/serve). Processed meats data from 2010, 2013, 2015 and 2017 were extracted from the Australian FoodSwitch database for the purpose of this study.

Products were categorized according to the Australian FoodSwitch database: bacon, canned meat, dried meat, frozen and chilled meat, meat burgers, salami and cured meats, sausages and hotdogs, and sliced meat. Frozen and chilled meat had the subcategories: coated/breaded frozen/chilled meat, meat with pastry, and uncoated frozen/chilled processed meat. Salami and cured meats were further divided into: cabanossi and twiggy sticks, chorizo, kransky, pancetta and prosciutto, polish salami, and salami. Sliced meats had the subcategories sliced ham, sliced luncheon meat, and sliced beef/chicken/pork/turkey.

A secondary categorization system was created to compare processed meat products to the FHD and HFP targets. The Australian FHD set maximum sodium targets for two categories of processed meats in 2011: bacon and ham/cured meat products, and emulsified luncheon meats. In 2012, the FHD set two further maximum sodium targets for wet savory pies and dry savory pies. The HFP drafted targets for eight processed meat categories: bacon, ham, delicatessen meats, frankfurts/saveloys, sausages, crumbed/battered meat/chicken, wet savory pies and dry savory pies. Products were included in this analysis if they met the government’s definitions for each food group [13,19].

Processed meats, defined under the Food Standards Code as a meat product that contains at least 300 g/kg of meat and has “either singly or in combination with other foods, undergone a method of processing other than boning, slicing, dicing, mincing or freezing”, and were included. Products were excluded if they did not meet this definition, there was no sodium displayed in mg/100 g on the package, or displayed erroneous data.

Sodium content was reported in mg/100 g of food. The total number of products, mean and standard deviation (SD), median and interquartile range (IQR), and range were determined for all processed meats, and grouped according to year and category/subcategory listed above. Products were also grouped according to presence or absence of a sodium target. The Shapiro–Wilk test was used to test for normality and differences in mean and median sodium levels between 2010, 2013, 2015 and 2017 were determined by conducting a one-way analysis of variance (ANOVA, with Scheffe’s post-hoc test) and Kruskal–Wallis tests (followed by Dunn’s test), respectively. The number and proportion of products meeting relevant sodium targets were also calculated across all years, and chi-square tests were used to determine differences in proportions meeting the targets between 2010 and 2017. Statistical analyses were conducted in Stata SE V13.0 for Windows (StataCorp LP, College Station, TX, USA) [20]. Alpha was set at a 0.05 significance level.

## 3. Results

A total of 2510 products were included in the analysis: 675 products from 2017, 633 from 2015, 783 from 2013 and 419 from 2010; 87% more products and three more product categories/subcategories were captured in 2013 compared to 2010, as illustrated in Table 1. Normality testing revealed that the sodium levels of processed meat overall, all categories and most subcategories were skewed. Only chorizo, sliced luncheon meats and uncoated frozen/chilled meats were normally distributed for all years. As such, median values were reported and non-parametric tests were conducted. Mean sodium levels can be found in Appendix A (Table A1).

### 3.1. Sodium Content of Processed Meats in 2017

In 2017, the median sodium content of processed meats was 775 mg/100 g (IQR 483–1080). There were large differences between the sodium content of processed meat categories. Dried meat had the highest sodium content per 100 g (median 1760 mg/100 g), followed by salami and cured meats (median 1475 mg/100 g). Frozen and chilled meats had the lowest sodium content (median 443 mg/100 g) followed by meat burgers (median 514 mg/100 g). Within some categories, large variation was evident (Table 1).

### 3.2. Trends in Sodium Levels from 2010 to 2017

The Dunn’s test following the Kruskal–Wallis test detected a 12% decrease in the median sodium levels of processed meats from 2010 to 2017 (880 to 775 mg/100 g; *p* = 0.001). However, this was not consistent across the years, with sodium levels decreasing by 18% from 2010 to 2013 (*p* = 0.001) followed by a 13% increase to 2015 (*p* = 0.106) and a 5% decrease to 2017 (*p* = 0.112). At the category level, a 13% decrease in the median sodium content of bacon (1205 to 1050 mg/100 g), and an 8% decrease in sliced meats (1075 to 986 mg/100 g) from 2010 to 2017 were determined (*p* < 0.001, *p* = 0.02, respectively). By contrast, the median sodium level of canned meat rose 46% from 540 to 786 mg/100 g (*p* = 0.006). The median sodium level decreased at the subcategory level for 4 subcategories, including a 10% decrease for meat with pastry from 456 to 410 mg/100 g (*p* = 0.017), a 19% reduction in pancetta and prosciutto from 2273 to 1835 mg/100 g (*p* = 0.016), an 11% reduction in sliced ham from 1160 to 1030 mg/100 g (*p* = 0.004) and a 16% reduction in sliced luncheon meats (*p* < 0.001; Table 1).

In contrast to the above, if we look at changes based on the mean, the Scheffe’s post hoc test following the ANOVA only revealed a significant reduction in the mean sodium levels of bacon (1259 mg/100 g to 1047 mg/100 g; *p* < 0.001) and sliced luncheon meats (980 mg/100 g to 809 mg/100 g; *p* = 0.002) from 2010 to 2017 (Appendix A (Table A1)). A sensitivity analysis determined that the new categories added in 2013 did not significantly affect the overall result (Appendix A (Table A2)).

### 3.3. Trends in Sodium Levels of Products with and Without Targets

There was an 11% reduction in the median sodium level of processed meats targeted by the FHD (*p* < 0.001), while there was no change in processed meats without a target (median difference 6%, *p* = 0.450). The median sodium level of processed meats with an FHD target (namely bacon, sliced ham, sliced luncheon meat and meat with pastry categories) was 1010 mg/100 g in 2010 and 898 mg/100 g in 2017. The median sodium level of all other processed meats was 765 mg/100 g in 2010, and 717 mg/100 g in 2017 (Table 1).

### 3.4. Trends in Sodium Levels of Products with and Without Targets

Only 35% of the products in 2017 were covered by an FHD target. Overall, 54% of products had sodium levels at or below their respective targets, including: 65% of ham/cured meat products, 65% of bacon, 51% of wet savory pastries and 29% of dry savory pastries, and 28% of emulsified luncheon meats (Table 2).

In 2010, 43% of available products had FHD targets, which dropped to 34% in 2013 and there has been no further change. Overall, the proportion of products at or below their respective target has increased from 23% in 2010 to 54% in 2017 (*p* < 0.001). By category, the proportion of bacon and ham/cured meat products at or below the 1090 mg/100 g target has increased (both *p* < 0.001) as well as the proportion of emulsified luncheon meats (*p* = 0.04); however, the proportion of savory pastries has remained unchanged (Table 2).

By contrast, the new HFP targets introduced in 2018 cover 444 of 675 (66%) products collected in 2017, across 8 processed meat categories; 35% of products were at or below their respective target, which has increased from 18% in 2010. In 2017, 49% of bacon products, 45% of ham, 40% of crumbed/battered meat products, 32% of wet savory pastries and 29% of dry savory pastries, 25% of frankfurts/saveloys, 21% of sausages and 19% of delicatessen meat were at or below their respective HFP targets (Table 2). The proportion of products at or below the target has continually increased for bacon, ham, and wet and dry savory pastries, where there were previous FHD targets. There has been no change for foods without FHD targets: frankfurts/saveloys, sausages or crumbed and battered meat and poultry. There has also been no change in the proportion of processed deli meats, a category that was partially covered by an FHD target.

## 4. Discussion

This research suggests that reductions in the sodium levels of processed meats have been made by the food industry in response to voluntary government targets. Overall, the sodium content of processed meats decreased by 12% from 2010 to 2017. However, further analysis revealed that the reductions were only for categories where the government had set targets under the FHD. This work builds on the findings by Trevena et al. [21], who determined that the proportion of bacon/ham/cured meat products meeting the FHD target increased between 2010 and 2013. The current study further showed that the proportion of these products meeting the targets continued to increase to almost two-thirds in 2017. Yet, the proportion of savory pastries meeting the targets has remained unchanged across the years, which may be due to the different timeframes for implementation. Irrespective of this, these results indicate that the initial setting of voluntary sodium targets under the FHD drove, at least in part, sodium reduction reformulation in processed meats in Australia.

Australia, like many countries, has chosen a voluntary approach to food supply sodium reduction. These have been successful in some countries, although others have not shown much progress. While two countries have legislated sodium reduction targets and others have chosen a combined mandatory and voluntary approach, voluntary approaches are the most widely implemented at present [22]. Our data indicate that the voluntary government targets in Australia drove sodium reformulation for only some processed meat categories, and there is evidence for this variable effect in other countries. In the UK between 2006 and 2011, significant reductions in the mean sodium content of bacon, sliced ham, cooked meat and frozen processed meat were observed; however there was no overall change for processed meat, nor meat with pastry products [23]. In Canada, the mean sodium levels in sausages/weiners, and fresh and frozen meat/poultry decreased under the Health Canada targets from 2010 to 2013, although the mean sodium content of other processed meat categories, including bacon and deli meats, remained unchanged [24]. In light of these variable results, continued and strengthened reformulation efforts in processed meats in Australia and globally are needed.

While the FHD target setting for processed meats drove sodium reformulation by the food industry, the limited coverage of products under the previous FHD targets constrained its potential impact. Processed meats are a significant contributor to daily sodium intake in the average Australian diet [15], and were thus chosen as a priority food category for target setting [12]. Yet, in 2010, less than half of products were captured by the targets, and from 2013 to 2017 only around one-third of products were covered. Magnusson and Reeve [25] further proposed that the limited impact may be due to variable industry participation. However, the companies which committed to sodium reformulation for processed meats and savory pies in 2009 represented 95% and 85% of the market share, respectively [19,26]. Thus, it is more likely that incomplete product coverage was the primary reason for the limited impact of the FHD targets.

Positively, a more comprehensive approach to target setting has been undertaken by the current government’s HFP, resulting in a higher product coverage and more stringent targets. The approach considered foods and beverages contributing ≥1% of sodium, sugar or saturated fat to the diet [27], a step which was noted by the Heart Foundation as a key consideration for food reformulation efforts in a recent review [28]. Other factors including feasibility, appropriateness of reformulation to reduce nutrient intake and available food supply data were also considered [27]. This approach resulted in the new draft HFP targets capturing two-thirds of processed meat products available in 2017, a great improvement on the previous FHD targets. Furthermore, this approach resulted in the draft HFP targets being set lower than the previous FHD targets for most processed meat categories, including ham, bacon, processed deli meats and wet savory pies, with only dry savory pies remaining the same. Applying these draft targets to the data revealed one-third of products were at or below their respective targets in 2017, whereas more than 50% of products were at or below the FHD targets. Taken together, given that there were only reductions in the sodium content of processed meats targeted by the FHD, the higher proportion of products captured by the HFP targets and the lower proportion of products already at or below the targets, this indicates large potential for sodium reduction across the processed meats category. This effort could be further strengthened by the addition of more processed meat categories, and plans for progressively lower targets in the future.

Food environment interventions, such as sodium reformulation, should be a component of a multifaceted sodium reduction program. Improving the food environment through sodium reformulation is recognized as a ‘best-buy’ strategy by the WHO, as it is cost-effective, affordable and feasible, and has population-wide impacts independent of individual change [29,30]. Furthermore, given that Australians are consuming almost double the recommended maximum daily amount of sodium [11], and processed meats contribute approximately 10% of average daily sodium intake [15], decreasing the sodium levels in processed meats through product reformulation could contribute to reducing population level daily sodium intake in Australia, and reducing the risk of associated adverse health implications. However, food environment interventions should be complemented by other initiatives, including consumer awareness campaigns to increase consumer knowledge and demand for healthier products, and front of pack labelling systems to drive reformulation and support healthier consumer choices [28]. Positively, the target setting undertaken by the HFP considered Australia’s voluntary front-of-pack labelling system, the Health Star Rating [31]. The majority of targets were set at a Health Star Rating baseline cut-point, providing an additional incentive for manufacturers to reformulate as they will likely achieve a higher score [27,32]. A national consumer awareness campaign should also be considered by the government, aiming to increase knowledge on the sodium content of all processed foods, awareness of the other health implications of frequently consuming processed meats (e.g., cancer [33]), and ultimately drive consumer demand for healthier products.

A strength of this study is the coverage of foods sampled. The majority of processed meat products were likely captured as data was collected on all processed meats sold in the four major supermarkets in Australia each survey year. This consistent sampling method, in combination with the rigorous quality assurance protocol employed [18], allowed for comparison between years. Notably, previous studies examining sodium levels in the Australian food supply have reported mean sodium levels and changes in mean sodium over time [21,34]. Another strength of this study is the use of medians to demonstrate change. Normality tests conducted revealed a skewed distribution of the data, and appropriate statistical tests (non-parametric tests) were subsequently performed. The findings suggested that the changes in the mean sodium over time resulted in conservative estimates of change, which is consistent with ANOVA tests being less powerful for skewed data. A limitation of the study was the lack of congruency between FoodSwitch categorization and the FHD targets, which required manual sorting. Further to this, due to the ambiguity of what the FHD classified as a cured meat, only bacon and ham were compared to the target. Additionally, three processed meat categories/subcategories were not captured in 2010, resulting in not all processed meat products being collected in that year.

## 5. Conclusions

Previous target setting attempts by the Australian government under the FHD has driven sodium reduction in processed meats. Promisingly, a higher proportion of processed meat products are captured by the proposed HFP targets, and a lower proportion of products are already at or below the proposed targets, compared to the FHD targets. Combined with the knowledge that the majority of sodium in the Australian population’s diet is from processed foods and approximately 10% from processed meats, these results demonstrate the large potential for future reformulation efforts in Australia to reduce population sodium intake.

## Figures and Tables

**Table 1:** Median sodium content, per category and subcategory, for processed meat.

	2010		2013		2015		2017		p-Value	
	n	Median (mg/100 g, IQR)	n	Median (mg/100 g, IQR)	n	Median (mg/100 g, IQR)	n	Median (mg/100 g, IQR)	Kruskal-Wallis	2010 vs. 2017 ^1^
**Processed meat**	419	880 (550–1200)	783	720 (480–1160)	633	816 (489–1100)	675	775 (483–1080)	0.008	0.001
**Bacon**	46	1205 (1090–1400)	52	1095 (1015–1320)	56	1085 (1000–1245)	63	1050 (946–1170)	<0.001	<0.001
**Canned meat**	53	540 (370–900)	60	674.5 (480–900)	37	867 (670–983)	41	785.7 (584–926)	0.002	0.006
**Dried meat**	NA	NA	31	2000 (1700–2495)	28	1830 (1672–2403)	24	1760 (1645–1860)	0.125	-
**Frozen and chilled meat**	56	456 (388–533)	241	453 (370–560)	185	440 (360–563)	220	442.5 (360–559)	0.720	-
Coated/breaded frozen/chilled meat	NA	NA	97	480 (360–599)	70	456 (360–605)	109	485 (380–610)	0.659	-
Meat with pastry	56	456 (388–533)	125	440 (375–521)	84	408.5 (360–484)	100	410 (355.5–496.5)	0.047	0.017
Uncoated frozen/chilled meat	NA	NA	19	488 (320–586)	31	574 (340–682)	11	353 (227–682)	0.228	-
**Meat burgers**	9	490 (350–692)	41	456 (350–555)	26	450 (390–555)	38	514 (425–603)	0.253	-
**Salami and cured meats**	70	1400 (1200–1550)	136	1410 (1334–1780)	97	1400 (1200–1680)	105	1475 (1300–1700)	0.113	-
Cabanossi and twiggy sticks	18	1200 (952–1330)	14	1200 (951–1330)	13	1200 (890–1330)	13	1060 (890–1480)	0.891	-
Chorizo	4	1145 (890–1480)	15	1130 (890–1630)	10	1070 (890–1500)	10	1275 (1050–1400)	0.923	-
Kransky	6	800 (760–930)	6	941 (890–985)	8	932 (876–983.5)	5	937 (927–985)	0.300	-
Pancetta and prosciutto	6	2273 (2190–2600)	30	1950 (1800–2330)	13	2260 (1980–2400)	22	1835 (1700–2200)	0.029	0.016
Polish salami	1	1020 (1020–1020)	1	789 (789–789)	1	1230 (1230–1230)	2	1300 (1300–1300)	NA	-
Salami	34	1434 (1400–1570)	70	1420 (1400–1680)	52	1490 (1400–1630)	53	1480 (1400–1680)	0.757	-
**Sausages and hotdogs**	99	680 (547–815)	113	660 (545–868)	80	784 (641.5–943.5)	91	719 (586–917)	0.017	0.158
Sausages	81	656 (538–720)	90	610 (529–720)	58	711.5 (590–794)	75	650 (569–840)	0.038	0.102
Hotdogs	18	1100 (950–1200)	23	947 (912–1120)	22	1110 (995–1400)	16	1060 (903.5–1140)	0.238	-
**Sliced meat (excl. salami, other cured meat)**	86	1075 (880–1200)	109	940 (815–1160)	124	919 (783–1050)	93	986 (831.5–1110)	<0.001	0.019
Sliced ham	43	1160 (1010–1320)	59	1150 (900–1200)	72	1010 (913.5–1160)	55	1030 (951–1140)	0.005	0.004
Sliced luncheon meat	13	971 (880–1010)	12	843 (787.5–963)	9	830 (783–838)	10	819.5 (732–838)	0.001	<0.001
Sliced beef, chicken, pork, turkey	30	895 (838–1100)	38	847 (665–940)	43	770 (530–847)	27	847 (720–1053)	0.010	0.164
**Processed meats with FHD target**	181	1010 (552–1200)	264	833 (456–1100)	244	920 (480–1090)	236	898 (472–1060)	<0.001	<0.001
**Processed meats without FHD target**	238	765 (548–1200)	519	692 (482–1350)	389	759 (508–1200)	439	717 (487–1190)	0.450	-

^1^*p*-value the result of Dunn’s test following the Kruskal-Wallis test when significant. FHD: Food and Health Dialogue; IQR: Interquartile Range.

**Table 2:** Comparison of sodium content of products against Food and Health Dialogue and Healthy Food Partnership Targets.

Target Name	FHD Target (mg/100 g)	*N* Meeting FHD Target (%)				*p*-Value ^1^	HFP Target (mg/100 g)	*N* Meeting HFP Target (%)				*p*-Value ^1^
		2010	2013	2015	2017			2010	2013	2015	2017	
Bacon	1090	11 (24)	19 (37)	28 (50)	41 (65)	<0.001	1005	7 (15)	12 (23)	18 (32)	31 (49)	<0.001
Ham/cured meat products	1090	12 (28)	29 (40)	49 (61)	39 (65)	<0.001	1005	9 (21)	26 (36)	36 (45)	27 (45)	0.012
Emulsified luncheon meats (processed deli meats)	830	3 (8)	6 (15)	6 (18)	8 (28)	0.035	720	5 (12)	11 (22)	17 (33)	7 (19)	0.363
Wet savory pastries	400	16 (33)	42 (48)	36 (51)	35 (51)	0.053	360	5 (10)	20 (23)	19 (27)	22 (32)	0.006
Dry savory pastries	500	0 (0)	1 (4)	2 (17)	6 (29)	0.111	500	0 (0)	1 (4)	2 (17)	6 (29)	0.111
Frankfurts and saveloys							900	3 (17)	3 (13)	2 (9)	4 (25)	0.548
Sausages							540	21 (28)	28 (33)	9 (16)	16 (21)	0.368
Crumbed and battered meat and poultry							450	N/A	42 (43)	34 (49)	44 (40)	-
Total		42 (23)	97 (35)	121 (48)	129 (54)	<0.001		50 (18)	143 (29)	137 (33)	157 (35)	<0.001

^1^*p*-value the result of chi-square test. FHD: Food and Health Dialogue; HFP: Healthy Food Partnership.

## Appendix A

**Table A1:** Mean sodium content, per category and subcategory, for processed meat.

	Mean (mg/100 g, SD)				p-Value	
	2010	2013	2015	2017	ANOVA	2010 vs. 2017 ^1^
**Processed meat**	920 (443)	878 (524)	897 (508)	857 (470)	0.183	-
**Bacon**	1259 (243)	1161 (245)	1152 (323)	1047 (167)	<0.001	<0.001
**Canned meat**	621 (270)	683 (227)	810 (210)	753 (254)	0.002	0.080
**Dried meat**	NA	1996 (556)	1936 (597)	1758 (337)	0.231	-
**Frozen and chilled meat**	474 (130)	476 (148)	468 (157)	465 (154)	0.865	-
Coated/breaded frozen/chilled meat	NA	503 (178)	482 (170)	497 (169)	0.729	-
Meat with pastry	474 (130)	457 (118)	428 (103)	434 (111)	0.058	-
Uncoated frozen/chilled meat	NA	463 (133)	544 (213)	419 (257)	0.15	-
**Meat burgers**	546 (244)	444 (152)	454 (135)	523 (161)	0.076	-
**Salami and cured meats**	1409 (429)	1523 (435)	1478 (454)	1493 (409)	0.339	-
Cabanossi and twiggy sticks	1162 (187)	1193 (271)	1129 (222)	1207 (311)	0.854	-
Chorizo	1185 (347)	1258 (525)	1153 (360)	1249 (259)	0.925	-
Kransky	834 (086)	978 (199)	928 (069)	891 (174)	0.332	-
Pancetta and prosciutto	2406 (508)	1924 (532)	2282 (425)	1875 (513)	0.026	0.170
Polish salami	1020 (.)	789 (.)	1230 (.)	1067 (330)	-	-
Salami	1486 (121)	1531 (192)	1515 (208)	1524 (230)	0.736	-
**Sausages and hotdogs**	721 (230)	718 (220)	830 (279)	764 (268)	0.008	0.638
Sausages	651 (153)	640 (157)	710 (198)	706 (242)	0.030	0.278
Hotdogs	1032 (265)	1023 (157)	1144 (208)	1040 (205)	0.199	-
**Sliced meat (excl. salami, other cured meat)**	1086 (358)	947 (316)	897 (272)	979 (271)	<0.001	0.140
Sliced ham	1192 (329)	1072 (261)	1020 (237)	1081 (234)	0.001	0.232
Sliced luncheon meat	980 (102)	882 (119)	818 (048)	809 (081)	<0.001	0.002
Sliced beef, chicken, pork, turkey	981 (427)	774 (351)	707 (239)	835 (298)	0.006	0.428
**Processed meats with FHD target**	953 (401)	811 (367)	856 (381)	812 (344)	0.0003	0.002
**Processed meats without FHD target**	896 (472)	913 (586)	923 (573)	881 (524)	0.7034	-
**Processed meats with HFP target**	868 (384)	710 (328)	754 (348)	717 (318)	<0.001	<0.001
**Processed meats without HFP target**	1026 (530)	1145 (652)	1161 (637)	1127 (583)	0.200	-

^1^*p*-value the result of Scheffe’s test following a one-way ANOVA test when significant. FHD: Food and Health Dialogue.

**Table A2:** Sensitivity analysis of processed meat category.

Year	Variable	Processed Meat	Processed Meat, Excluding Categories Not Available in 2010	*p*-Value ^2^
2010	Number of Products	419	419	1.00
	Mean (mg/100 g, SD)	920 (443)	920 (443)	
	Median (mg/100 g, IQR)	880 (550–1200)	880 (550–1200)	
	Range (mg/100 g)	120–3300	120–3300	
2013	Number of Products	783	636	0.55
	Mean (mg/100 g, SD)	878 (524)	894 (480)	
	Median (mg/100 g, IQR)	720 (480–1160)	813 (510–1185)	
	Range (mg/100 g)	60–2920	60–2830	
2015	Number of Products	633	504	0.45
	Mean (mg/100 g, SD)	897 (508)	919 (455)	
	Median (mg/100 g, IQR)	816 (489–1100)	885 (558–1160)	
	Range (mg/100 g)	124–3300	124–-3300	
2017	Number of Products	675	531	0.12
	Mean (mg/100 g, SD)	857 (470)	899 (466)	
	Median (mg/100 g, IQR)	775 (483–1080)	864 (530–1120)	
	Range (mg/100 g)	74–3200	82–3200	
*p*-value ^1^	2010 vs. 2017	0.23	0.92	

^1^*p*-value the result of Scheffe’s post-hoc test following a one way analysis of variance (ANOVA). ^2^*p*-value the result of an independent *t*-test.
